# The Quality of Life Supports Model as a Vehicle for Implementing Rights

**DOI:** 10.3390/bs13050365

**Published:** 2023-04-28

**Authors:** Lucía Morán, Laura E. Gómez, Miguel Ángel Verdugo, Robert L. Schalock

**Affiliations:** 1 Department of Psychology, University of Oviedo, 33003 Oviedo, Spain; moranlucia@uniovi.es; 2 Institute on Community Integration (INICO) and Department of Personality, Assessment and Psychological Treatments, University of Salamanca, 37005 Salamanca, Spain; verdugo@usal.es; 3 Department of Psychology, Hastings College, Hastings, NE 68901, USA; rschalock@centurylink.net

**Keywords:** quality of life, supports, quality of life supports model, Convention on the Rights of Persons with Disabilities, CRPD, sustainable development goals, intellectual disability, developmental disabilities, intellectual development disorder

## Abstract

The Quality of Life Supports Model (QOLSM) is emerging as a new framework that is applicable to people with disabilities in general, but specially to people with intellectual and developmental disabilities (IDD). The aim of this conceptual paper is twofold. Firstly, it aims to show the overlap between the QOLSM and the Convention on the Rights of People with Disabilities (CRPD), highlighting how the former can be used to address many of the goals and rights embedded in the latter. Secondly, the article seeks to illustrate the connection between these two frameworks and highlight the importance of acknowledging and measuring the rights of people with IDD. Therefore, we posit that the new #Rights4MeToo scale is ideal for: (a) providing accessible means and opportunities for people with IDD to identify and communicate their needs regarding their rights; (b) enhancing the supports and services that families and professionals provide to them; and (c) guiding organizations and policies to identify strengths and needs in relation to rights and quality of life. We also discuss future research needs and summarize the main findings of this article, highlighting its implications for practice and research.

## 1. Introduction

Over the last 50+ years, important changes in the field of intellectual and developmental disabilities (IDD) have been catalysts for the emergence of the new shared citizenship paradigm. The shared citizenship paradigm is one that envisions, supports, and requires the engagement and full participation of people with disabilities, but especially people with IDD, as equal, respected, valued, participating, and contributing members in every aspect of society [1]. This paradigm is currently guiding the development of individualized supports and services, organizational strategies, and policies related to IDD. This paradigm is also very relevant because it provides a framework for evaluation, application, and research. 

It is based on contemporary values and beliefs that recognize the rights of people with IDD to participate fully in all aspects of life. It considers contextual factors that influence the manifestation of IDD, and aims to reduce barriers to shared citizenship, meet needs, and support optimal health and functioning throughout life. In other words, the shared citizenship paradigm aims to improve the lives of people with IDD by promoting their active participation in society and enhancing their valued outcomes. 

The overall goals of the paradigm are to further advance and focus on people with IDD as active agents in the mainstream of life and in change processes. Schalock et al. [2] enumerated four core factors that have driven this paradigm:

1. A holistic approach to IDD that reinforces a whole-person approach to services and supports (taking into account biomedical, psychoeducational, sociocultural, and justice perspectives);
2. A contextual model of human functioning that explains disability as resulting from the interaction between the person and their natural, built, cultural, and social environments;
3. Person-centered implementation strategies that represent best practices and drive evidence-based practices, that are based on current best evidence and that use reliable and valid methods derived from a clearly articulated and empirically validated model;
4. Disability rights principles, such as belonging, equity, inclusion, empowerment, participation, and self-determination.

Actually, the shared citizenship paradigm is reflected in international civil and human rights covenants, such as the Convention on the Rights of People with Disabilities (CRPD). In this respect, the CRPD [3] serves as a mechanism for promoting, protecting, and monitoring the fulfillment of rights and shared citizenship of people with disability, and therefore for recognizing, quantifying, and making visible the serious and complex situations of disadvantage and discrimination faced by this population, especially by people with IDD [4,5,6,7]. 

In practice, however, the implementation of the CRPD is not without its challenges. One of the multiple reasons for this is the abstract nature of some of the CRPD content. For example, the CRPD includes a number of broad principles and goals such as “full and effective participation and inclusion in society” or “respecting the dignity of people with disabilities”. While these goals and principles are important and provide a useful framework for disability rights advocacy, they can be difficult to operationalize in practice: What specific actions or policies are necessary to ensure full and effective participation in society? What does dignity mean and how should it be upheld in practice? This can make it difficult to determine whether specific policies or practices are consistent with the principles of the CRPD, making implementation and evaluation difficult. For this reason, there is a clear need to define specific measurable indicators to assess progress [8]. 

Several authors [9,10,11,12] have suggested that the quality of life (QOL) construct provides a valid framework from which to operationalize, measure, and implement the CRPD articles. QOL provides a way to measure and evaluate the effectiveness of disability policies and services in a holistic and person-centered manner. By focusing on domains such as social inclusion, personal development and well-being, QOL offers a nuanced and comprehensive view of the experiences of people with disabilities, translating abstract principles and goals into measurable personal outcomes. While the CRPD provides a framework and set of principles for the rights and inclusion of people with disabilities, the QOL construct offers a way to evaluate the effectiveness of policies, programs, and services in promoting well-being and fulfillment. 

On the one hand, the QOL paradigm is based on the idea that QOL is a multidimensional construct that involves a subjective experience that is influenced by a broad range of domains, including personal and environmental factors. The supports paradigm, on the other hand, focuses on the importance of providing people with disabilities with the necessary strategies and resources to prevent or mitigate the disability or its effects (e.g., personal assistance, assistive technology, prosthetics, life-long learning opportunities, reasonable accommodations, employment opportunities, mental health promotion programs). 

An adequate provision of individualized supports is essential for enhancing the QOL of people with IDD. Appropriate supports can help them to overcome barriers to full participation in society, increase their independence and autonomy, and promote greater well-being and satisfaction with life. For example, providing access to assistive technology, such as communication devices, can help them to overcome communication barriers, enhancing their ability to participate in social and community activities. For this reason, the QOL construct has recently been merged with the supports construct to create the Quality of Life Supports Model (QOLSM). The QOLSM aims to provide a useful framework for policy development, supports provision, organization transformation, systems change, and outcome evaluation [13]. 

The purpose of this article is twofold. Firstly, it aims to show the overlap between the QOLSM and the CRPD. Secondly, the article introduces a new tool, the #Rights4MeToo scale, which was initially designed for people with IDD. This tool enables the measurement of two key concepts highlighted in the QOLSM—QOL and rights—in a practical and quantitative way. The article seeks to illustrate the connection between the two frameworks and highlight the importance of acknowledging and measuring the rights of people with IDD. It is crucial to address the inequalities faced by them in terms of their rights and QOL so as to ensure that they have equal opportunities to participate fully in society and achieve their full potential. Finally, we discuss future research needs, and conclude by summarizing the main findings of the article and highlighting its implications for practice and research.

## 2. The QOLSM

After 25 years of parallel paths, the constructs of QOL and individualized supports have been merged to create the QOLSM [13,14,15]. The QOLSM defends a community approach in which the focus is placed on the characteristics of the context, and the success of interventions is measured in terms of QOL. On the one hand, QOL is a global concept centered on the person; it provides information about what is important in an individual’s life and what outcomes must be achieved (for example, emotional well-being: reducing high levels of anxiety). On the other hand, supports are centered on how these outcomes can be achieved (for example, through a psychological intervention such as positive behavior support and facilitating alternative and adaptive modes of communication to help them express themselves). 

The QOLSM is a holistic and integrated approach focused on the rights, self-determination, equity, and inclusion of people with disabilities. This new approach emphasizes individualized supports in inclusive environments, and promotes the evaluation of personal outcomes to implement evidence-based practices. Below, we summarize the four essential components of the QOLSM as well as its multiple uses.

### 2.1. Essential Components of the QOLSM

The four essential components of the QOLSM are core values, individual and family QOL domains, systems of supports, and facilitating conditions.

#### 2.1.1. Core Values

Core values stem from the beliefs and assumptions that people hold about individuals with IDD, and their individual worth and potential. These core values guide policies and practices regarding people with IDD and their roles in society [13]. The core values that QOLSM brings together are the recognition of the human and legal rights of people with IDD [16,17,18,19] enshrined in the CRPD, the capacity and potential of people with IDD to grow and develop [20,21], the emphasis on self-determination, social inclusion and equity [22,23,24], and the commitment to address people’s support needs and foster opportunities to enhance individual functioning and personal well-being [25,26]. These values are fundamental to the QOLSM, and are essential for promoting the QOL of people with IDD.

In this sense, one of the core values emphasized by QOLSM is the recognition of the human and legal rights promulgated in the CRPD. This includes the right to be treated with dignity and respect, the right to make decisions about their own lives, and the right to participate fully in society. By acknowledging these rights, the QOLSM promotes the empowerment of people with IDD and their full inclusion in society.

#### 2.1.2. Individual and Family QOL Domains

Individual and family QOL domains are understood as a set of factors that reflect a clear approach centered on the individual or family, and the application principles related to equity, empowerment, self-determination, inclusion, and valued outcomes. The domains also provide a framework for using the QOLSM for person-centered outcome evaluation, and supports provision, systems change, and organization transformation [27].

For example, the individual QOL model proposed by Schalock and Verdugo [28] has gained wide acceptance in the field of IDD, but also in other groups of vulnerable people at risk of social disadvantage. This model has a great amount of empirical evidence regarding its validity, and it is commonly used internationally by IDD support organizations and professionals [29,30]. According to this model, QOL is a multidimensional concept composed of eight core domains (i.e., social inclusion, self-determination, rights, interpersonal relationships, personal development, emotional well-being, material well-being, physical well-being) that reflect the degree to which people have experiences that are meaningful for them. 

With regard to family QOL, for instance, the theoretical proposal by Zuna et al. [31] conceptualizes FQOL as a dynamic sense of well-being in the family, collectively and subjectively defined and informed by its members, in which individual- and family-level needs interact. These authors propose four concepts whose functioning inside the family system can affect family QOL: family unit concepts, individual member concepts, performance concepts, and systemic concepts. 

#### 2.1.3. Systems of Supports

Systems of supports provide the framework for improving functioning and well-being. As we mentioned before, they are a broad set of resources and strategies that prevent or mitigate the impact of a disability, but they also help promote development, education, and interests. 

The supports paradigm helps identify the types and amount of support that the person needs. This information is then used to group people with similar support needs together (i.e., subclassification goals) and create support strategies that are tailored to their needs (i.e., aligning supports needs to support strategies). The supports model also helps to identify the different components that make up a system of supports, which can then be put into action to provide the necessary support to people with IDD.

A commonly used grouping of the elements of systems of supports includes choice and personal autonomy, generic and specialized supports, and inclusive environments [25]. Generic supports are broad-based and can be applied across a range of situations and individuals. They are typically available to everyone, such as access to public transportation, general education, and community services. Specialized supports are more targeted and specific to the needs of a particular person or group of people. They are designed to address specific challenges or barriers that a person may face and may require specialized training or expertise to provide (e.g., speech therapy, occupational therapy, behavior support). The provision of generic and specialized supports allows an individualized and comprehensive approach to supporting people with IDD [14,32]. 

#### 2.1.4. Facilitating Conditions

Facilitating conditions are contextual factors that influence the successful application of the QOLSM [13]. These contextual factors are influenced by—and interact with—properties of the micro- (individual), meso- (interpersonal or organizational level), and macrosystem (societal level) [33,34,35]. 

QOL-facilitating conditions refer to the conditions that promote the QOL of people with IDD. For example, promoting a sense of belonging within the community, maximizing their abilities and opportunities, providing safe and secure environments, and respecting their personal goals and aspirations [13]. Support-facilitating conditions, on the other hand, refer to the factors that facilitate the provision of effective supports. These include understanding the person’s support needs, making sure that their personal goals are assessed and addressed, providing accessible and appropriate supports, ensuring that support providers are knowledgeable and competent, and coordinating and managing supports effectively [13].

### 2.2. Uses of the QOLSM

The QOLSM is a theory-based and professionally sound framework for supports provision and person-centered outcome evaluation (microsystem), organization transformation (mesosystem), and systems change (macrosystem) [33,34,35].

#### 2.2.1. Supports Provision

The essential purpose of supports provision is to reduce the discrepancy between an individual’s functional limitations and the demands of their environment, thereby enhancing their functioning and personal well-being. Relatives, primary caregivers, and professionals are the main support-providers. Three strategies are the most applicable to these individuals who provide supports: (1) an emphasis on QOL, (2) the provision of supports related to choice and personal autonomy opportunities, and (3) the use of generic supports that are available to all and can be provided by multiple support providers. These three strategies provide connections, interactions, and facilitating conditions.

#### 2.2.2. Person-Centered Outcome Evaluation 

The purpose of person-centered evaluation is to employ the knowledge, skills, and resources of a partnership to measure and effectively use outcome information to enhance personal well-being, increase transparency, facilitate accountability, and expand understanding [36]. The QOLSM provides a framework for person-centered outcome evaluation, given that it aligns core values with a modern understanding of IDD, individualized supports, valued outcomes, and meaningful impacts. This approach to outcome evaluation involves a collaborative partnership between an individual, a human service organization or system, and a team comprising the individual and their various formal and informal support providers.

#### 2.2.3. Organization Transformation

Organizations that apply one or more components of the QOLSM develop new ways of thinking and implement new policies and practices related to their service delivery system, thereby transforming themselves in significant ways [14,15]. Examples include maximizing the person’s capabilities; being committed to the goals that are important to the person or family; conceptualizing supports as a bridge between “what is” and “what can be”; believing that with appropriate individualized supports over a sustained period, an individual’s QOL and functioning generally will improve; implementing policies and practices that include the availability and accessibility of supports; and conducting QOL-focused outcome evaluation. 

#### 2.2.4. Systems Change

The QOLSM provides a framework to produce the systems change envisioned in the CRPD [14,15]. As stated by Mittler [8], the CRPD articles incorporate the principles and values embedded in the QOL concept, and the CRPD goals encourage signatories to make “reasonable accommodation” in their support delivery systems to enable people with disabilities and their families to exercise their rights and experience a higher QOL. Thus, the CRPD is a commitment to the human rights of people with disabilities, so that no one is left behind. This value and principle of “leaving no one behind” is shared with the United Nations 17 Sustainable Development Goals (SDGs) [37], actions that all countries must take to reduce inequality, recognizing that the inclusion of people with disability is fundamental to sustainable development. Systems change can be based on the alignment of QOL domains, CRPD articles, SDGs, and systems of supports elements [12].

## 3. The #Rights4MeToo Scale

As mentioned above, there is a close relationship between the CRPD articles, the QOLSM, and the SDGs [12]. All three undertake to prevent anyone from being relegated to a non-citizenship status, and they are also committed to enhancing human rights and the inclusion of people with IDD into the mainstream of life [38]. For this reason, there is a need for QOLSM-based measurement instruments that demonstrate adequate evidence of reliability and validity. One such instrument is the #Rights4MeToo scale [4,12,39], a tool for assessing the rights promulgated in the CRPD for people with IDD, based on the QOLSM and capturing many aspects of the SDGs. 

Actually, the scale allows for the operationalization of the QOLSM by providing a way to measure the concepts outlined in the model in a concrete and quantitative manner. The field-test version of the #Rights4MeToo scale provides a set of 153 items structured around Schalock and Verdugo’s eight QOL domains. Then, within these domains, the items are further mapped to the relevant articles of the CRPD. In other words, this instrument provides a set of specific items that can be used to assess QOL (one of the main constructs of the QOLSM) and allows for the identification of supports (the other main construct of the QOLSM) that the person with IDD needs in order to fully enjoy and effectively exercise their rights as a full citizen (one of the core principles of the QOLSM). 

The process to develop and provide evidence of the reliability and validity of the instrument has been progressive. Verdugo et al. [9] first laid the theoretical foundations on the close relationship between the CRPD and QOL, by aligning the CRPD articles to the eight QOL domains. The next study, conducted by Lombardi et al. [10], focused on reaching an international consensus on the relationship between core QOL indicators and the CRPD articles. Through a Delphi study with 153 experts (including people with IDD, family members, professionals, researchers, and lawyers) from 10 countries, more than 80 cross-culturally validated QOL indicators were obtained to operationalize the CRPD. Subsequently, Gómez et al. [11] carried out a systematic review of the scientific literature. They identified dozens of indicators and personal outcomes related to the CRPD articles promulgating specific rights, and then mapped them to the eight QOL domains. Next, Gómez et al. [40] consulted 32 experts (including professionals, relatives of people with IDD, and researchers) to select 153 items that obtained the highest scores in terms of suitability, importance, and clarity. These items made up the field-test version of the #Rights4MeToo scale. 

Once this pool of items was agreed, the next steps focused on adapting the field-test version of the scale to an easy-to-read format and having the items validated by people with IDD. This process involved three self-advocates with IDD, a psychologist who acted as a facilitator in the validation sessions, and a professional. The professional was in charge of the initial adaptation of the items, instructions, and response format, and then for the layout of the final version of the instrument. The process was completed over five sessions, each lasting approximately 2 h. In addition to validating the easy-read version of the items, the self-advocates had the opportunity to suggest new items that had not initially been considered. They also took part in a qualitative study about their knowledge of the CRPD and about what rights they thought were—or were not—respected for people with IDD [4]. Finally, an electronic version of the #Rights4MeToo scale was developed for computers and tablets, along with an instruction guide and an explanatory how-to video.

The #Rights4MeToo scale is addressed to (a) people with IDD aged 12 years or above and (b) proxies (e.g., close people such as relatives and professionals) who have known the person with IDD for at least 6 months and who are aged 4 years or above. Items are therefore presented in the first person when the person with IDD answers for themselves (i.e., self-report), and in the third person when a proxy answers for the person with IDD (i.e., hetero-report). When people with IDD respond (self-report version), the recommendation is to complete the questionnaire over two or three 45 min sessions due to the length of the instrument. However, when the respondents are professionals, family members, and legal representatives (hetero-report version), the scale is usually completed in a single session lasting approximately 20 min.

**Figure 1:**
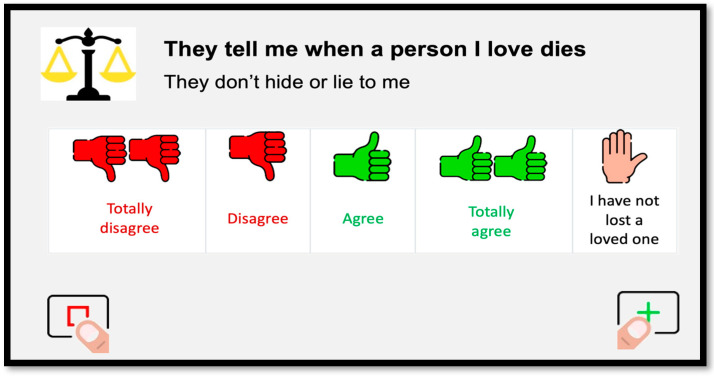
Example of an item, explanation, and answer format in the self-report version of the #Rights4MeToo scale.

Items are presented one by one on the computer or tablet screen, and the person must click on the “next” button (icon with a finger on the + symbol) to progress to the next item. If no answer option is selected, an error message appears telling the person that they must choose an answer to continue. If the person wants to take a break and continue at another time, they can click on “exit and save” (icon with a finger on the square symbol). As shown in Figure 1, the items are short statements that are displayed in bold, followed by a brief explanation to facilitate understanding, and preceded by an icon representing the QOL domain to which the item belongs. Each item is presented in a Likert format with four answer options (i.e., “totally disagree”, “disagree”, “agree”, “totally agree”) that are presented in text (colored in red when referring to disagreement and in green when referring to agreement) and accompanied by icons (i.e., hand/s with thumbs up or thumbs down) in the same colors. A few items include a fifth option, which corresponds to “not applicable”. These are also represented by a hand icon in a different color, accompanied by a statement relevant to the situation being described. For example, for the item “They tell me when a person I love dies” (that is, “they don’t hide or lie to me”), there is the option to select “I have not lost a loved one”. 

**Figure 2:**
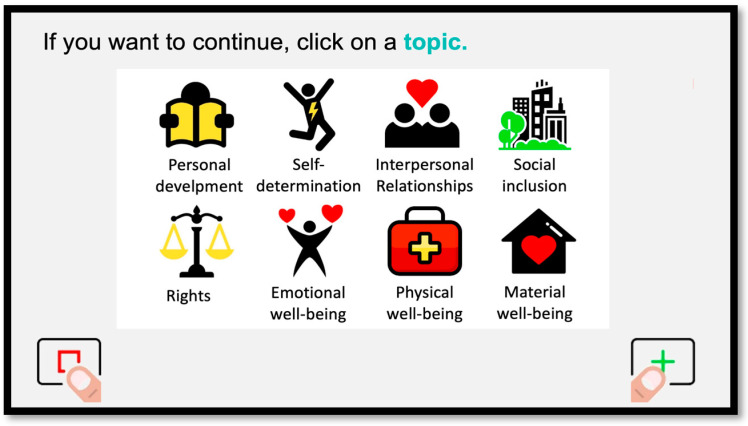
Example of the QOL domain selection screen in the #Rights4MeToo scale.

In addition, the web version of this tool allows the person to customize their experience. The respondent can choose the order in which they want to complete the QOL domains (Figure 2). Further, the wording and content of the items will change to match the characteristics of the respondent. For example, the items are written with she/her pronouns if the person indicates that she identifies as a woman or a girl. Similarly, items related to employment are not presented if the respondent is a minor, and items related to school and education are shown instead. 

**Figure 3:**
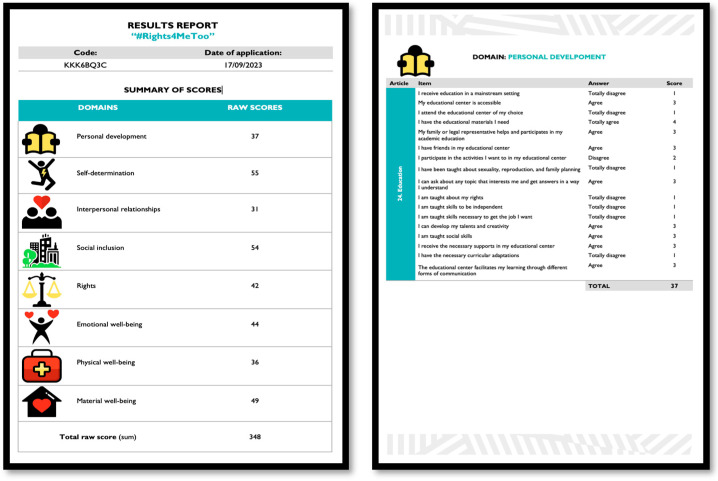
Sample excerpt from a results report.

The web application also includes a feature to download a report that automatically calculates the total scores in the eight QOL domains, and shows the specific responses to each of the items structured around the relevant CRPD articles (Figure 3). The obtained scores are interpreted by taking into account that higher scores indicate greater enjoyment of QOL and greater exercise of rights by the person with IDD. The QOL domains and the articles of the CRPD that obtained lower scores would be priority areas for providing supports. In this sense, the final version of the scale will provide a representation of the standard scores obtained in each QOL domain and CRPD article in a profile that will graphically illustrate the strengths and needs of the person in terms of rights. 

When several evaluations from different perspectives are conducted for the same person with IDD (i.e., self-report, report of a relative, report of a direct support professional), priority should be given to the perspective of the person with IDD themselves, but it is also recommended to triangulate the information by analyzing similarities and differences in the information provided by the different informants. The aim is to conduct a comprehensive evaluation and provide the most appropriate individualized supports to maximize the person’s chances of exercising their full citizenship.

The #Rights4MeToo scale can be considered an innovative tool that fills a void and addresses an urgent need. It can be used to (a) providing accessible means and opportunities for people with IDD to identify and easily communicate their needs regarding their rights and daily situations involving discrimination or noncompliance with what has been ratified in the CRPD (i.e., microsystem); (b) serve as a tool that professionals and family members can use to detect strengths and weaknesses in relation to rights, thereby improving the support they provide to people with IDD (i.e., microsystem); and (c) evaluate and monitor the effectiveness of the programs and supports implemented by organizations in terms of rights (i.e., mesosystem), as well as guide and monitor public policies (i.e., macrosystem).

## 4. Future Research Needs

Two kinds of studies are still needed (1) to improve the knowledge concerning the QOLSM, and (2) to provide evidences about the validity and usefulness of the #Rights4MeToo scale. 

On the one hand, there is a need for theoretical articles to further develop and operationalize the QOLSM. There is a need for studies that can provide a more comprehensive understanding of its underlying principles and mechanisms. These studies could help us identify areas where the model may be improved, as well as provide a basis for developing more effective interventions and supports that can enhance the QOL of people with IDD. In addition, theoretical studies could help establish a stronger empirical foundation for the model, by testing its assumptions and exploring its relationships with other concepts and constructs. While there are limited published studies on it, the QOLSM has been widely used and adopted by practitioners and researchers in the field of IDD, which speaks to its relevance and potential usefulness. Future studies can contribute to the ongoing development and evolution of the QOLSM, and help ensure its continued relevance and usefulness in guiding the provision of supports and promoting QOL.

On the other hand, we think that the #Rights4MeToo scale has great potential as a tool for promoting the full exercise of rights and enhancing the QOL for people with IDD. The scale can serve as a valuable tool for identifying and addressing the needs of people with IDD in relation to their rights, and can inform the development of tailored support and interventions. Additionally, the scale’s focus on the intersection of IDD and experiences of discrimination, violence and abuse can help raise awareness and promote action to prevent such situations, and provide adequate supports. By providing a standardized, evidence-based approach to assessing the rights of people with IDD, the scale can contribute to advancing the field and promoting greater inclusion and equity worldwide for this population.

However, although there is already considerable evidence of its content-based validity [4,11,40] and reliability [4], the #Rights4MeToo scale is still in the validation process. The scale has been responded to by more than 1200 people in Spain. Their responses will be used to select the most reliable items and to provide evidence regarding its validity based on its internal structure. We will also study the role and influence of important variables such as age, level of supports needs or gender, and we will examine the relationships between the different perspectives (i.e., people with IDD, professionals, and family members). 

In the future, another line of research should involve adapting the scale for use in other countries, which would allow for cross-cultural studies and comparisons. Another potential line of research could be analyzing the scale’s utility and psychometric properties in other specific groups with disabilities, such as people with Autism Spectrum Disorders, Down’s syndrome, cerebral palsy, rare diseases, acquired brain injury, dementia, or other conditions and disorders.

## 5. Conclusions

This paper highlights the need to continue advancing on the effective implementation of the rights of people with IDD, relying on the QOLSM as the ideal framework for translating such abstract concepts as equity, empowerment, self-determination, inclusion, and valued outcomes into evidence-based practices and policies. 

People with disabilities, including people with IDD, have the right to live in the community, to receive inclusive and adequate education, to access quality healthcare services, to work, to be treated with dignity and respect, to have a partner and a family, to participate in the cultural and social life of the community, to access the same resources and opportunities as any other person, and to be a citizen with full rights. The rights of people with disabilities, including people with IDD, are inalienable and unconditional. 

However, people with IDD usually face significant inequalities in terms of their rights and QOL, such as limited access to healthcare, education, and employment opportunities, as well as social isolation and stigma. To improve their full citizenship and QOL, it is essential to address these inequalities through policy and practice changes, such as promoting inclusive education, and ensuring that healthcare providers are trained to meet the specific needs of people with IDD. Respecting and exercising their rights is not only a matter of justice and equity, but it is also a key factor for sustainable development and the building of a more inclusive and supportive society. We must work together to ensure that all people, including those with IDD, have the same opportunities, can achieve their full potential and fully participate in community life. 

In this proposal, with the #Rights4MeToo scale, the QOLSM is used to assess the effective fulfillment of goals and rights embedded in the CRPD, by (a) empowering and giving an active role to people with IDD to know and defend their rights, providing a tool and opportunities to communicate their needs regarding rights in a meaningful way; (b) enhancing the supports and services that families and professionals provide to people with IDD; and (c) guiding organizations and policies to identify the strengths and needs in relation to rights, QOL, and supports. Hence, a major strength of the operationalization of the QOLSM using the #Rights4MeToo scale is the measurement of personal and valued outcomes, its focus on context, and the power to reflect the perspective of people with IDD, and what is truly important to enhance their quality of life and personal well-being.

## Data Availability

No new data were created or analyzed in this study. Data sharing is not applicable to this article.
